# Combating COVID-19 and its co-infection by *Aspergillus tamarii* SP73-EGY using in vitro and in silico Studies

**DOI:** 10.1038/s41598-024-77854-0

**Published:** 2025-01-03

**Authors:** Eman Abdelsalam, Amal Mosad Ibrahim, Ahmed A. El-Rashedy, Mohamed S. Abdel-Aziz, Omnia Kutkat, Faten K. Abd EL-Hady

**Affiliations:** 1 Chemistry of Natural and Microbial Products Department, Pharmaceutical and Drug Industries Research Institute, National Research Centre, 33 El Buhouth St, Dokki-Giza, Egypt; 2 Department of Microbial Chemistry, National Research Centre, Giza, 12622 Egypt; 3 Centre of Scientific Excellence for Influenza Viruses, Water Pollution Research Department, Environment Research and Climate Change Institute, National Research Centre, Giza, 12622 Egypt

**Keywords:** Antimicrobials, Bacteria, Fungi, Virology, Drug discovery, Microbiology, Chemistry

## Abstract

The COVID-19 pandemic has caused significant mortality and morbidity for millions of people. Severe Acute Respiratory Syndrome-2 (SARS-CoV-2) virus is capable of causing severe and fatal diseases. We evaluated the antiviral properties of *Aspergillus tamarii* SP73-EGY isolate extract against low pathogenic coronavirus (229E), Adeno-7- and Herpes-2 viruses. The extract showed a high selectivity index (SI = 43.4) and a significant inhibition of 229E (IC_50_ = 8.205 μg/ml). It was stronger than the drug control, remdesivir (IC_50_ = 38.2 μg/ml, SI = 7.29). However, the extract showed minimal efficacy against Adeno-7- and Herpes-2-Viruses (IC_50_ = 22.52, 47.79 μg/ml, and SI = 6.75, 5.08, respectively). It exhibited profound efficacy against the highly pathogenic SARS-CoV-2 (IC_50_ = 8.306 μg/ml, SI = 42.2). Kojic acid, the primary component of the extract, showed substantial antiviral activity against SARS-CoV-2 (IC_50_ = 23.4 μg/ml, SI = 5.6), Remdesivir (IC_50_ = 4.55 μg/ml, SI = 61.45). Therefore, the extract demonstrated the most notable antiviral characteristics against coronavirus infection. Co-infecting microorganisms may contribute to immune system deterioration and airway injury caused by SARS-CoV-2. The extract showed significant efficacy against *E. coli* and *P. aeruginosa****,*** with an inhibition range of 3.5–10 mm at a concentration of 200 mg/ml. A molecular docking study showed that hexadecanoic, Kojic, octanoic acids, and 4(4-Methylbenzylidene)cyclohexane-1,3-dione have stronger binding affinity to the SARS-CoV-2 M^pro^ than Remdesivir. Molecular dynamics simulations were employed to examine the structural stability and flexibility of these complexes. This confirmed the high binding affinities of Kojic acid and 4(4-Methylbenzylidene)cyclohexane-1,3-dione, thereby proving their potential as novel anti-SARS-CoV-2.

**Supplementary Information:**

The online version contains supplementary material available at 10.1038/s41598-024-77854-0.

## Introduction

Coronavirus 2, commonly referred to as SARS-CoV-2, is a coronavirus characterized by a non-segmented positive-stranded RNA genome. The WHO identifies SARS-CoV-2 as the etiological agent of the COVID-19 pandemic^1^, leading to over two hundred million fatalities and more than 276,000,000 confirmed cases. Currently, as of the latest update on July 5, 2023, the cumulative count of vaccine doses delivered is 1,727,843,186. The duration and effectiveness of protection against SARS-CoV-2 infection and vaccination are essential factors in guiding pandemic policy plans, especially in deciding the best timing for delivering booster doses of the vaccine^1^.

### Coronavirus strains

Multiple strains of the coronavirus have been distinguished by researchers, namely the severe acute respiratory syndrome coronavirus (SARS CoV), the Middle East respiratory syndrome coronavirus (MERS CoV), and the severe acute respiratory syndrome coronavirus 2 (SARS CoV-2). Their capacity to cause serious and potentially fatal diseases is considerable^2^. The serious acute respiratory syndrome coronavirus-2 (SARS-CoV-2) is the causative agent of COVID-19 and has a genetic resemblance of around 70% with SARS-CoV^3^. Upon entering the respiratory system of mammals via particles, it proceeds to infect the epithelial cells of the trachea, bronchi, and alveoli, so enabling the spread of the virus to other persons^4^. Based on their genomes and serogroups, coronaviruses are classified into four genera: (α-CoVs), (β-CoVs), (γ-CoVs), and (Η-CoVs). Furthermore, scientists have classified HKU1, HCoV-NL63, HCoV-229E, HCoV-OC43, MERS-CoV, SARS-CoV, and SARS-CoV-2 as coronaviruses with the ability to induce illness. A fundamental attribute of viruses is their inherent susceptibility to mutation, which poses significant difficulties in the management of diseases and ultimately leads to a worldwide epidemic^5^.

### Microbial co-infecting

The presence of co-infecting microorganisms has the potential to contribute to the deterioration of the immune system and the injury of the airways that are associated with respiratory infections caused by SARS-CoV-2. The COVID-19 co-infections have been associated with a limited number of species, including viruses, bacteria, fungi, and archaea. Even though we don’t fully understand how co-infecting microorganisms cause SARS-CoV-2, they may be responsible for harming the immune system, the airways that carry air, the death of neurons, the growth of goblet cells, changes in mucus production, decreased ciliary beat regularity, function, and clearance, and less oxygen transfer^6^.

The increasing prevalence of resistance to current therapeutic interventions necessitates the development of highly effective antiviral drugs targeting SARS-CoV-2. To date, Ongoing research has been undertaken to explore new pharmaceutical interventions for severe acute respiratory syndrome infections resulting from Coronavirus 2 (SARS-CoV-2).

### Marine sponge-associated fungi

Marine sponge-associated fungi exhibit biodiverse activities; however, their antiviral effectiveness against SARS-CoV-2 remains undetermined. The discovery of marine bioactivities as potential pharmaceuticals has led to the initiation of several research programs aimed at developing novel chemical moieties with innovative biochemical pathways for pharmaceutical corporations^7^.

Marine organisms encompass a significant proportion, exceeding two-thirds, of the Earth’s surface, rendering them a substantial supply of novel compounds that act as drugs^8^. Marine-associated fungi originate bioactive molecules with potent antiviral properties^9^. A wide range of secondary metabolites derived from marine organisms have been discovered to possess various beneficial properties, including anti-inflammatory, anticancer, antibacterial, antiviral, antimalarial, and antioxidant activities^10^.

Natural bioactive compounds derived from marine sources may be used as medicines and potential SARS-CoV-2 inhibitors to improve COVID-19 treatment. They exhibit remarkable utility in combating medication-sensitive diseases due to their unique chemical structures and diverse biodiversity, which introduce novel mechanisms of action^11^.

According to reports, *Aspergillus* species play a crucial role in the production of antiviral medicines^12^. Kojic acid molecule of *Aspergillus* sp. is beneficial as a depigmenting agent^13^ and possesses antifungal, antibacterial^14^, antioxidant, and other properties^15^.

### In silico study

Numerous studies have attempted to use active ingredients derived from natural sources to combat COVID-19 illness to guide their actions, utilizing in in silico investigations^16^. When developing SARS-CoV-2 antagonists and describing the biological properties and the potential of tested compounds to attach to significant therapeutic targets, in silico techniques such as molecular docking are frequently employed.

In this study, we reported for the first-time antiviral activity, particularly anti-SARS-CoV-2, from ethyl acetate extract of the fungus *Aspergillus tamarii* isolate SP73-EGY, associated with the sponge *Amphimedon viridis*, Red Sea, Egypt, using in vitro, in silico*,* and molecular dynamic investigations for the secondary metabolites identified by GC/MS analysis.

## Results and discussions

Ongoing research is conducted to discover new, highly effective medications for severe acute respiratory syndrome infections caused by the coronavirus-2 (SARS-CoV-2).

Despite various biological activities, marine sponge-associated fungi’s antiviral actions against SARS-CoV-2 are still ambiguous.

Furthermore, our ongoing investigation focused on the biomedical applications and chemical constituents of fungi found in marine sponges ^17,18^**.** The present study examined the NEW and extremely significant anti-COVID-19 extract and its new anti-COVID-19 components; "Kojic acid and 4(4-Methylbenzylidene)-cyclohexane-1,3-dione," from *Aspergillus tamarii* isolate SP73-EGY associated to the sponge "*Amphimedon viridis*, Red Sea, Egypt". The observed impact of the extract against the Herpes-2 and Adeno-7 viruses was merely marginal.

### Molecular identification of isolated fungal strain

**Fig. 1:**
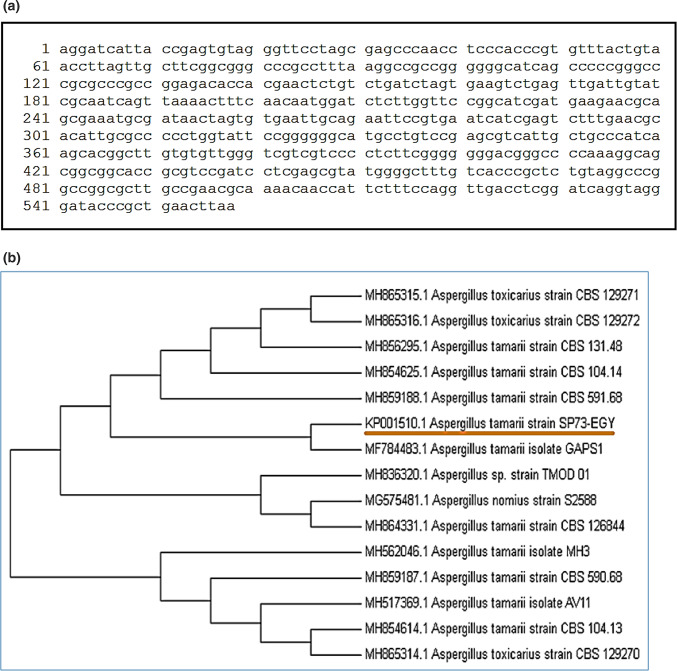
(**a**) Aligned sequence data of 18rDNA amplified from the fungal strain SP73-EGY. (**b**) Phylogenetic tree showing relationship of strain (SP73) with other related fungal species retrieved from GenBank based on their sequence homologies of 18SrRNA.

The 18SrRNA method was used to identify the fungal isolate (SP73) associated to the sponge "*Amphimedon viridis"*. Using the Basic Local Alignment Search Tool (BLAST; http://www.ncbi.nlm.nih.gov), relatives were identified by comparison to rRNA genes in the National Center for Biotechnology Information (NCBI) Gen Bank database, the aligned sequence data of 18rDNA amplified from the fungal strain SP73-EGY had been represented in (Fig. 1a) and a phylogenetic tree was created (Fig. 1b). The fungal strain was kept in serve in the Culture Collection of Microorganisms of the Microbial Chemistry Department, NRC, Egypt. The strain’s sequences were delivered to GenBank under the name "*Aspergillus tamarii* isolate SP73-EGY" with the GenBank accession number (KP001510). The internal transcribed spacer (ITS) region (1–5) of ribosomal DNA (rDNA) was used as a target region within the RNA gene cluster. ITS1 and ITS4 primers were used to amplify the ITS’s. Polymerase chain reaction (PCR) and sequencing were efficient, accurate, and rapid for microbial identification^18–20^.

### GC/MS analysis

In addition to a high concentration of kojic acid (65%), the GC/MS analysis showed that the extract contained 24 other substances. The sample primarily contains two compounds: Table 1 shows that the main compounds are 4(4-Methylbenzyl-idene)-cyclohexane-1,3-dione (15%) and 1,11-Bis(methoxycarbonyl-ethenyl)-10,2-dihydroxy-cycloeicosane (5.7%), with other compounds showing different percentages.

**Table 1:** Chemical composition assessed by GC/MS analysis for *A. tamarii* isolate SP73-EGY extract.

No	Rt	COMPOUNDS	MW	MF	Area %
***Fatty acids***					
1	5.92	D-Lactic acid	234	C_9_H_22_O_3_Si_2_	0.58
2	9.71	4-Hydroxybutanoic acid	248	C_10_H_24_O_3_Si_2_	0.73
3	14.87	Octanoic acid	216	C_11_H_24_O_2_Si	0.42
4	17. 70	Succinic acid	262	C_10_H_22_O_4_Si_2_	1.13
5	18.90	2,3-DihydroxyPropanoic acid	322	C_12_H_30_O_4_Si_3_	0.27
6	23.0	Valeric acid-5-methoxy	204	C_9_H_20_O_3_Si	0.13
7	23.73	3,4-dihydroxy butanoic acid	336	C_13_H_32_O_4_Si_3_	0.27
8	23.97	2-isopropyl-3-hydroxy-2-butenoic acid	288	C_13_H_28_O_3_Si_2_	1.07
9	37. 27	Azelaic acid	332	C_15_H_32_O_4_Si_2_	0.07
10	44.80	Hexadecanoic acid (Palmitic acid)	328	C_19_H_40_O_2_Si	0.84
11	49.74	Oleic acid	354	C_21_H_42_O_2_Si	1.18
12	50.46	Octadecanoic acid (Stearic acid)	356	C_21_H_44_O_2_Si	0.14
***Phenolic and halogenated compounds***					
13	13.86	Nicotinamide	194	C_9_H_14_N_2_OSi	1.52
14	21.53	4-Chlorobenzoic acid	228	C_10_H_13_ClO_2_Si	2.07
15	26.27	4(4-Methylbenzylidene)cyclohexane-1,3-dione	214	C_14_H_14_O_2_	15.21
16	31.49	Benzoic acid-3,4-dimethoxy	254	C_12_H_18_O_4_Si	0.13
17	34.70	N,N,N',N'-Tetramethyl-p-phenylphosphonic-diamide	268	C_14_H_25_N_2_OP	1.3
***Others***					
18	5.83	propylene glycol	220	C_9_H_24_O_2_Si_2_	1.67
19	14.05	Diethylene glycol	250	C_10_H_26_O_3_Si_2_	0.80
20	14.19	2,3-Dihydroxybutane	234	C_10_H_26_O_2_Si_2_	1.50
21	24.70	2,4-Di(cyclopenta-2,4-dien1ylidene)-1,1,3,3-tetramethyl cyclobutane	236	C_18_H_20_	0.10
22	25.45	2-Aminoquinolino[8,7e] [1,2,4]triazine-4-oxide	213	C_10_H_7_N_5_O	0.10
23	25.49	Cyclohexanone-3-carboxylic acid	214	C_10_H_18_O_3_Si	0.48
24	32.93	Kojic acid	286	C_12_H_22_O_4_Si_2_	22.33
	35.70	Kojic acid (tautomer)*	286	C_12_H_22_O_4_Si_2_	42.85
25	58.50	1,11-Bis(methoxycarbonyl-ethenyl)-10,2-dihydroxy-cycloeicosane	480	C_28_H_48_O_6_	5.65

Rt = Retention time. MW = molecular weight, MF = Molecular Formula. * Kojic acid (tautomer) = [kojic acid has two ring hydrogen atoms, which lead to a large number of possible tautomer^59^].

A total of 24 components were identified through the application of GC/MS analysis. Among these components, twelve fatty acids of varying chain lengths were detected, including D-lactic acid (0.58%), octanoic acid (0.42%), succinic acid (1.13%), hexadecanoic acid (0.84%), and oleic acid (1.18%).

There have been reports of some GC/MS-identified compounds having antiviral properties. For instance, both D and L-lactic acid have demonstrated the ability to decrease the titers of MNV and H1N1 viruses by approximately 3.25 log units and 2.5 log units, respectively^21^. Octanoic acid has the ability to render the human immunodeficiency virus, pseudorabies virus, bovine viral diarrhea virus, and Sindbis virus; all are lipid-encased viruses^22^. Succinic acid has been proposed as a potential antiviral treatment for COVID-19^23^. Succinate inhibits the production of IFNβ- by macrophages infected with the vesicular stomatitis virus (VSV)^24^.

Fatty acids have a significant impact on virus infection. The fatty acids exhibiting the highest antiviral efficacy are those with the greatest number of carbon chains^25^. Oleic acid inactivates the viral envelope at 10 µg/mL, while, at a concentration of 50 µg/mL, it completely eradicates any viral particles that may be present. The fatty acids efficiently degrade the bilayer lipid sheath^25^. According to research, the OH group of hexadecanoic acid has been shown to be hazardous to cell protoplasm, damage cell walls, denaturize proteins in the cytoplasm, and form hydrogen bonds on enzyme active sites^26^.

**Fig. 2:**
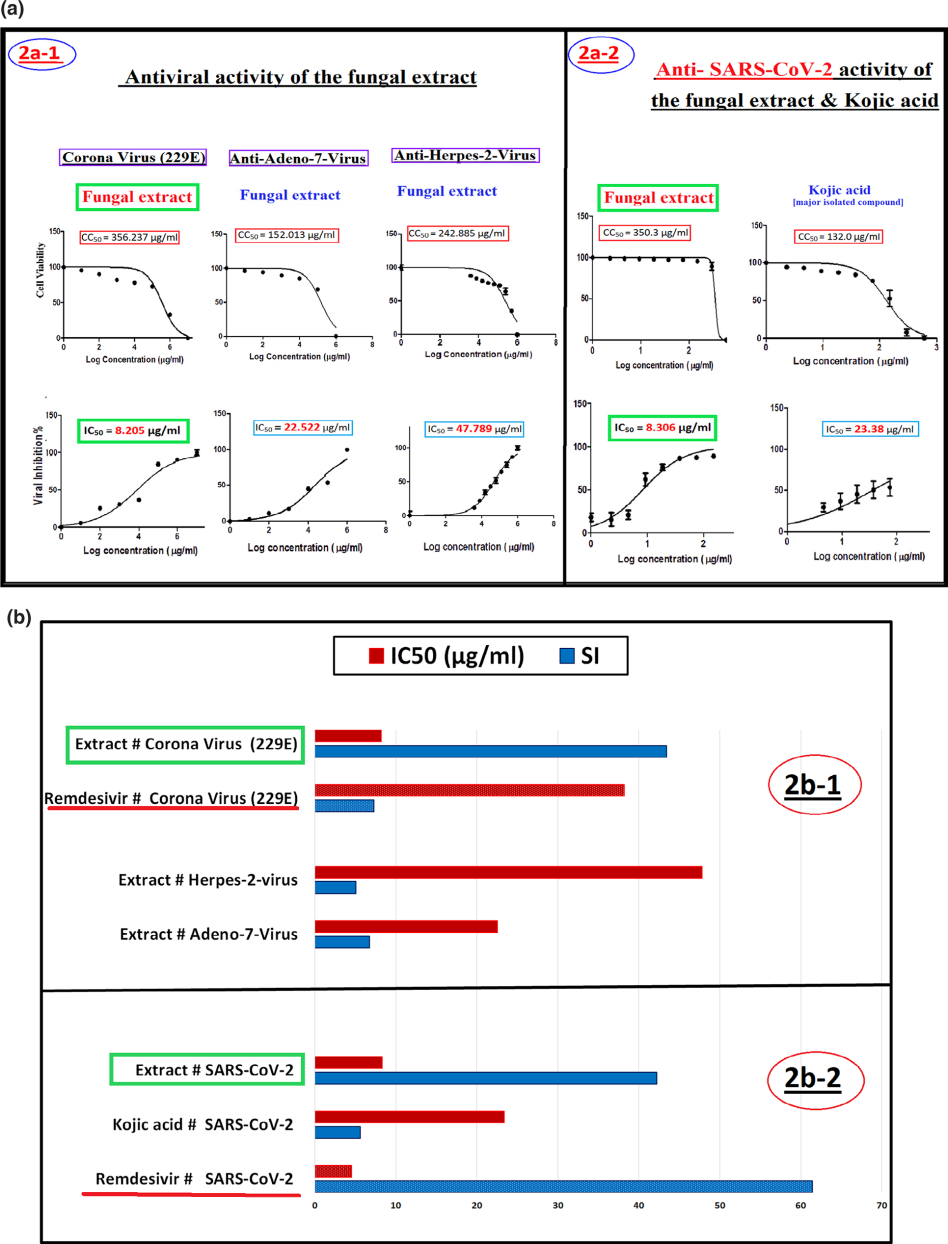
(**a**) (2a-1) Antiviral activities (CC_50_, IC_50_) of the fungal extract against low pathogenic Corona Virus (229E), Adeno-7-Virus and Herpes-2-virus. (2a-2) Antiviral activities (CC_50_, IC_50_) of the fungal extract and kojic acid against (SARS-CoV-2) virus. **(b)** (2b-1) Antiviral activities (IC_50_ and SI) of the extract against [Corona virus (229E) & Remdesivir]. The extract against Herpes-2-virus and Adeno-7-virus. (2b-2) Antiviral activities (IC_50_ and SI) of the extract, kojic acid, and Remdesivir against (SARS-CoV-2) virus.

Characterization of the major isolated compound (65%). The compound exhibited significant novel antiviral efficacy against the highly pathogenic SARS-CoV-2 with IC_50_ = 23.4 μg/ml and SI = 5.6 (Fig. 2a, b). The GC/MS test (Table 1) of the ethyl acetate extract showed that it contained a major compound (65%); its spectroscopic data were compiled (Table 2).

**Table 2:**
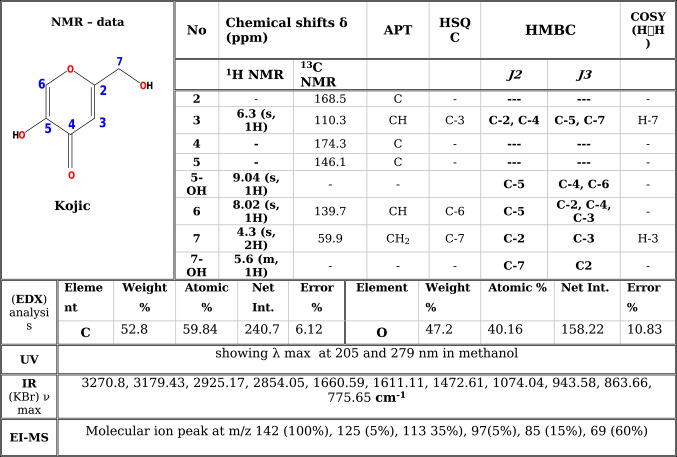
All spectroscopic data of isolated major compound (Kojic acid).

The compound was isolated as a white crystal. (EDX) analysis revealed the presence of carbon and oxygen and the absence of nitrogen, phosphorus, halogens, and sulfur. IR (KBr) ν max; 3270.8, 3179.43 cm^–1^ (OH), 2925.17 cm^-1^, 2854.05 cm^-1^ (aliphatic-CH), 1660.59 cm^-1^ (cyclic -C = O), 1611.11 cm^-1^ (C = C), 1472.61 cm^-1^ (deformation of—CH_2_), 1074.04 cm^-1^ (cyclic C–O–C). EI-MS; Its molecular ion peak at m/z 142 (100%) and fragment ions at m/z; 125 (5%), 113 35%), 97(5%), 85 (15%), 69 (60%) (Table 2). 1D and 2D-NMR data revealed that ^1^H -NMR δ_H_: 9.04 (s, 1H, 5-OH), δ_H_ 6.3 (s, 1H, H-3), δ_H_ 8.02 (s, 1H, H-6), and protons δ_H_ 4.3 (s, 2H, H-7). ^13^C-NMR (APT) δ_C_:( 174.3 (C = O), δ_C_ 168.5(C-2) and δ_C_ 146.1(C-5) δ_C_ 139.7(C-6), δ_C_ 110.3 (C-3) and δ_C_ 59.9 (C-7). The ^1^H-^1^H correlation spectroscopy (COSY) data from H-3 to H-7 and from H-7 to H-3. The connectivity of each carbon with the corresponding proton/s was confirmed by the (HSQC) experiment. the ^1^H-^13^C heteronuclear multiple bond correlations (HMBC) from H-6 to C-5, C-2, and C-4, while H-3 to C-2, C-7, C-5 and C-4, H-7 to C-2, C-3. Furthermore, proton at 9.04 (5-OH) to (C-4), (C-6), (C-5). Proton at 5.6 (7-OH) to (C-2) and (C-7) suggested the presence of kojic acid (Table 2). The previous findings indicated a kojic acid structure that closely aligned with the previously described structure^27^.

### Antiviral activities

#### (A) Antiviral activity of the fungal extract against

Human corona virus (229E), human Adeno-7-Virus and Herpes-2 virus

The antiviral activity of the fungal extract was investigated against Low Pathogenic human Corona Virus (229E), human Adeno-7-Virus and herpes-2 virus. The *A. tamarii* extract inhibited the low pathogenic coronavirus strain (229E), with an IC_50_ value of 8.205 μg/ml and safety index (SI) of 43.4. The antiviral activity of remdesivir as a pharmacological control was shown to be somewhat restricted, with a specific inhibitory concentration (SI) of 7.29 and an IC_50_ value of 38.2 μg/ml.

In contrast, *A. tamarii* exhibited a relatively low safety index equal 6.75 when evaluated for its antiviral efficacy against human Adeno-7-Virus (Ad7). Additionally, the safety index equal 5.08 against the Herpes-2 virus in the study (Fig. 2a-1, b-1). Consequently, *A. tamarii had low antiviral activity against* human Adeno-7-Virus (Ad7) and Herpes simplex type -2 virus.

### (B) Anti-SARS-CoV-2 activity of the fungal extract and its major compound (kojic acid)

According to the results depicted in (Fig. 2a-1, b-1), *A. tamarii* extract showed high significant antiviral activity against Low Pathogenic Corona Virus (229E) with a selectivity index (SI, 43.4). These data prompted us to investigate this extract against the highly pathogenic SARS-CoV-2 virus.

With an IC_**50**_ value of 8.306 μg/ml and a selectivity index (SI) of 42.2, the extract showed strong activity against the very dangerous SARS-CoV-2. Furthermore, the major compound isolated from the extract, consisting of 65% "kojic acid," displayed notable antiviral activity against SARS-CoV-2, with an IC_50_ value of 23.4 μg/ml and SI of 5.6, compared with the antiviral activity of remdesivir as a drug control against SARS-CoV-2 with (SI) 61.45 and IC_50_ = 4.55 μg/ml (Fig. 2a-2, b-2).

Finally, the results of the antiviral activities conducted on four viruses (Fig. 2a, b) indicate that the extract derived from *A. tamarii* exhibited the most significant antiviral effects against coronaviruses because, the virus did not able to infect the cells and make propagation after incubation with extract for 1 h before infect the cell because of efficacy of the extract on spike protein receptors which presented on the envelope so, it had direct action to destroy or deformation of the virus surface proteins (virucidal activity)^28^**.**

Therefore, based on the results obtained, the fungal extract subjected to testing exhibits promising potential as a viable candidate for conducting additional experiments on its efficacy against the coronavirus.

The results obtained suggest that *A. tamarii* antiviral activity might be partially due to a direct interaction of the compounds in the extract with the viral envelope, given the effect on highly pathogenic (SARS‑CoV-2) and low pathogenic Corona Virus (229E). The findings of our study enabled us to elucidate the inhibitory potential of *A. tamarii* in the context of viral infection, particularly SARS-CoV-2, the causative agent of the global COVID-19 pandemic.

### Antimicrobial activities

Co-infections with different bacteria and fungi are common in SARS-CoV-2 patients, which has a significant impact on the severity and fatality rates of COVID-19. There is a paucity of studies on co-infecting species, their interactions, and how they eventually interact with hosts.

**Fig. 3:**
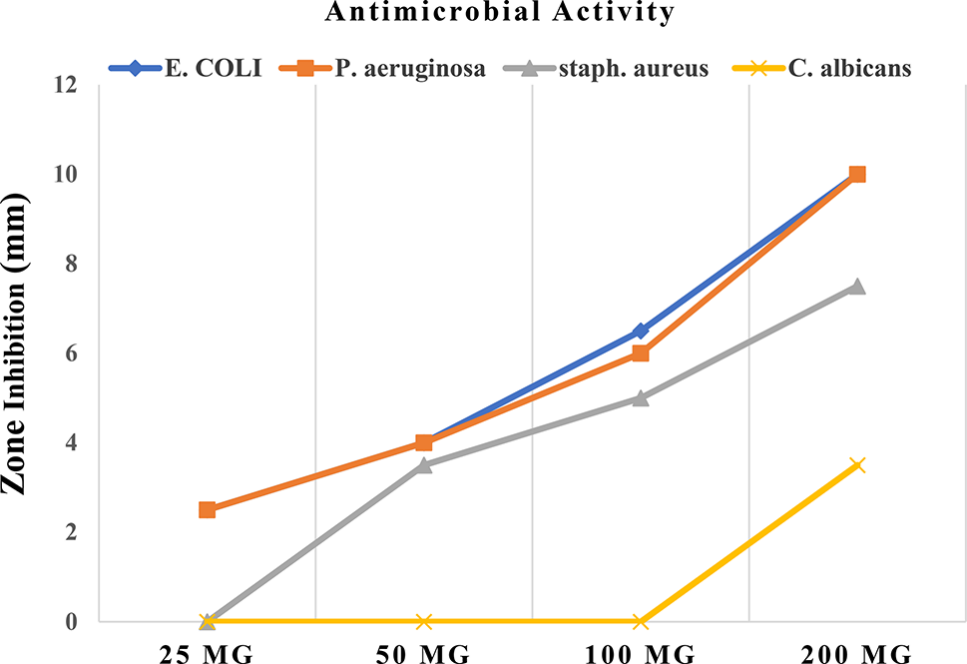
Antimicrobial activities of the fungal extract against *E. coli, P. aeruginosa, Staph. aureus and C. albicans*; at concentrations of 25–200 mg/mL.

The ethyl acetate extract of *A. tamarii* was tested for its antimicrobial activity using the Cup-plate method on harmful Gram-positive, Gram-negative, and yeast bacteria*.* The results indicated that the ethyl acetate extract exhibited significant antibacterial efficacy against Gram-negative bacteria while demonstrating limited antibacterial effectiveness against Gram-positive bacteria. Furthermore, no discernible impact on yeast was observed. The inhibition’s diameter varied from 3.5 to 10 mm. The extract demonstrated significant bacteriostatic impact on both *E. coli* and *P. aeruginosa*, moderate activity against *S. aureus*, and no activity against *C. albicans*. These findings suggest that the extract exhibits enhanced efficacy at a concentration of 200 mg/ml, as depicted in (Fig. 3).

Since these bacteria and fungi are proven to be harmful and resistant to many antibiotics, the results found are significant due to the cause of hospital infections. *P. aeruginosa* and *E. coli* showed a high level of intrinsic resistance to the majority of antibiotics^29,30^.

This study identified 12 short and long chain fatty acids using the GC/MS analysis (Table 1). It was found that simple natural fats with antibacterial capabilities, including fatty acids, can kill both Gram-positive and Gram-negative bacteria as well as coated viruses, fungi, and yeast^31^. Numerous fatty acids have antibacterial and antifungal properties, and by directly interacting with cells, they may be able to alter immunological reactions. Oleic acid was more efficient against *P. aeruginosa* infection because of its liposomal composition^32^. Hexadecenoic and octadecanoic acids may have antibacterial and antifungal properties^33^.

### In silico study

#### Molecular dynamic and system stability

**Fig. 4:**
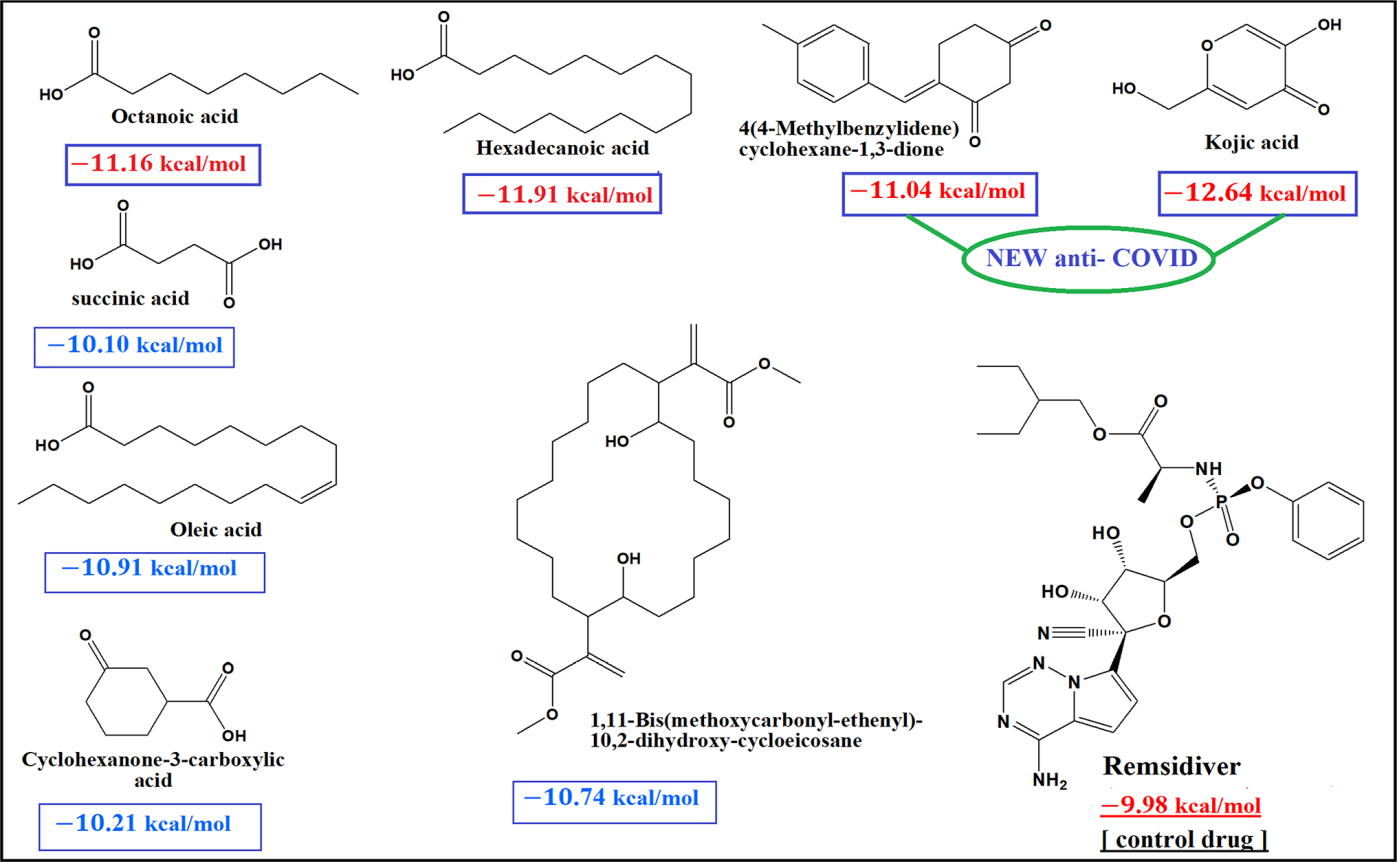
Structures of the high anti-COVID-19 compounds identified by GC/MS analysis and summary of molecular operating environment (MOE) docking results (binding energy kcal/mol) for the fungal extract highly significant identified compounds (-11.04 to -12.64 kcal/mol) and high active compounds (-10.10 to -10.91 kcal/mol) comparing to Remsidiver (-9.98 kcal/mol)). The graph pointed to the NEW anti-COVID-19 compounds {kojic acid and 4(4-Methylbenzylidene)- cyclohexane-1,3-dione}.

A molecular docking simulation was carried out to predict the performance of the most active that are afforded from the Molecular docking study for the 25 compounds identified with GC/MS analysis . These compounds include hexadecanoic acid, Kojic acid, octanoic acid, and 4(4-Methylbenzylidene)cyclohexane-1,3-dione had ahigher docking score compared to Ramisidiver^34^. According to Nguyen et al., the key residues in the binding pockets of of inhibitor Ramisidiver in M^pro^ are His163, Ser144, and Leu141, and non-bonded contacts are associated with Glu166, Cys145, Met165, Gln189, Arg188, Asp187, His41, Met49, Thr26, Leu27, Thr45, and Thr25 residues^34^.These areas are similar to the hexadecanoic acid, Kojic acid, octanoic acid, and 4(4-Methylbenzylidene)cyclohexane-1,3-dione binding pocket, indicating that hexadecanoic acid, Kojic acid, octanoic acid, and 4(4-Methylbenzylidene)cyclohexane-1,3-dione and remdesivir bind to similar active site of the main protease of SARS-CoV-2 (M^pro^) protein. (Table S1, Fig. S1) Simulations showed that the protein was stable upon binding of hexadecanoic acid, Kojic acid, octanoic acid, and 4(4-Methylbenzylidene)cyclohexane-1,3-dione to the active site of protein^35,36^ (Fig. 4). The validation of system stability is essential to trace disrupted motions and avoid artifacts that may develop during the simulation.

**Fig. 5:**
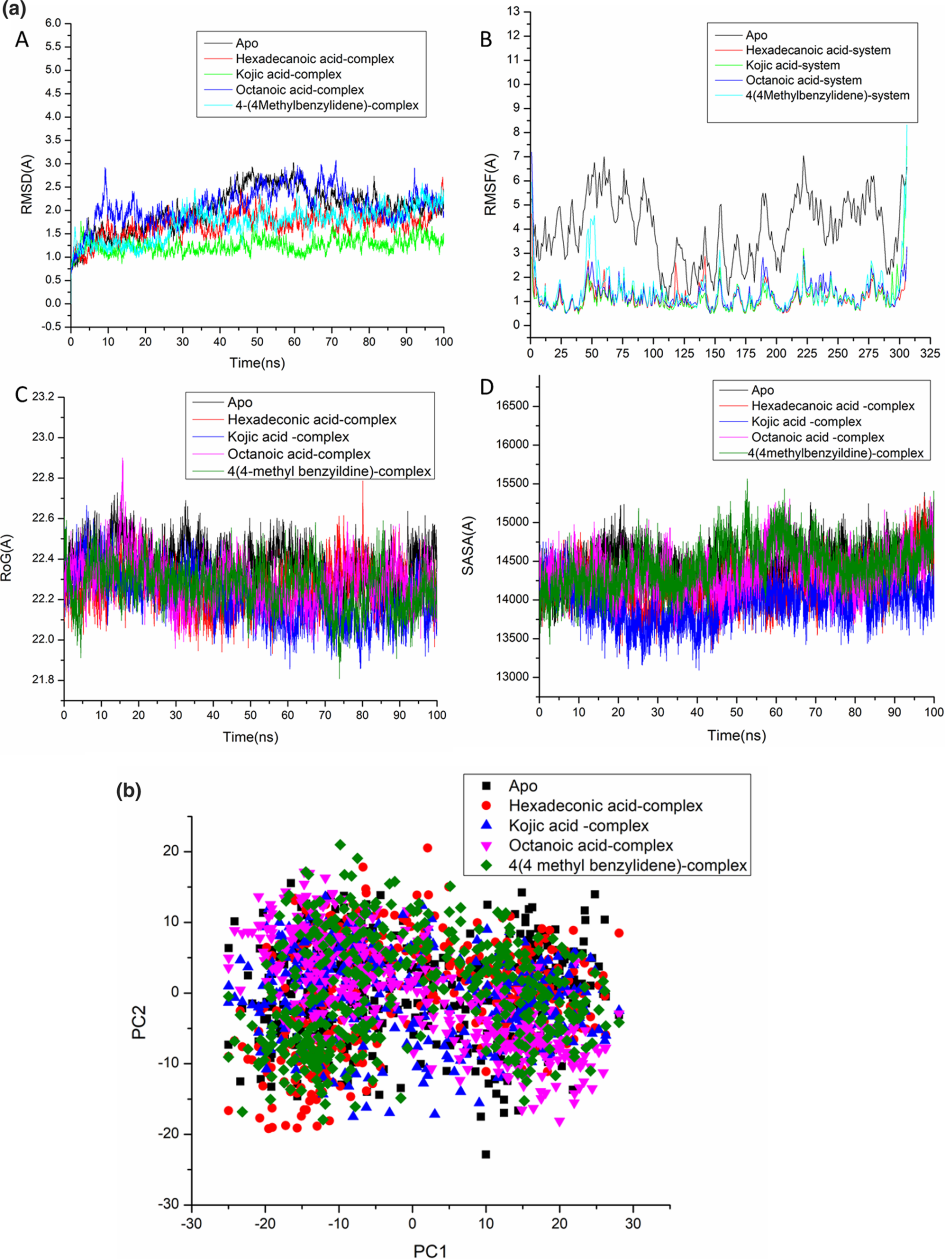

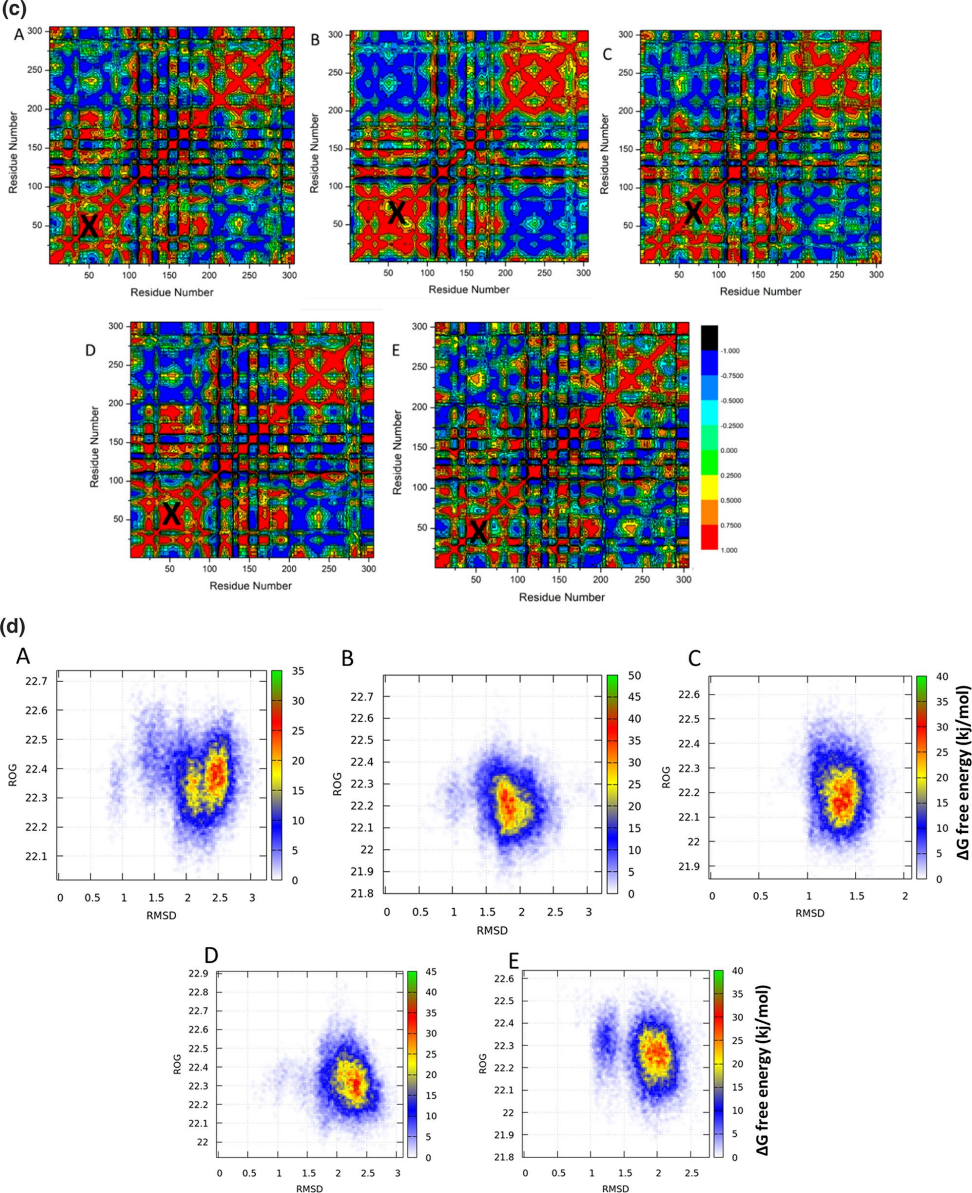

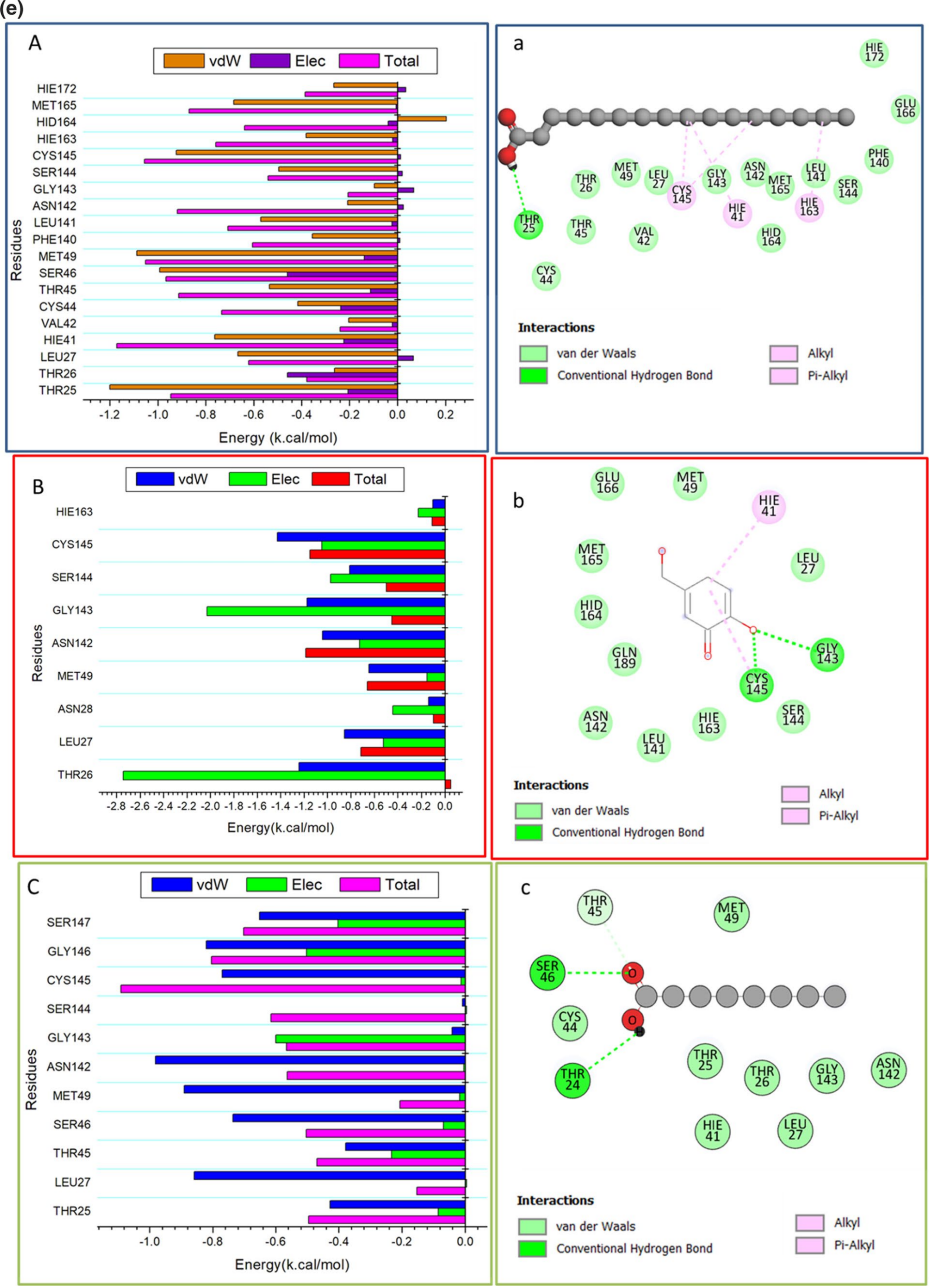

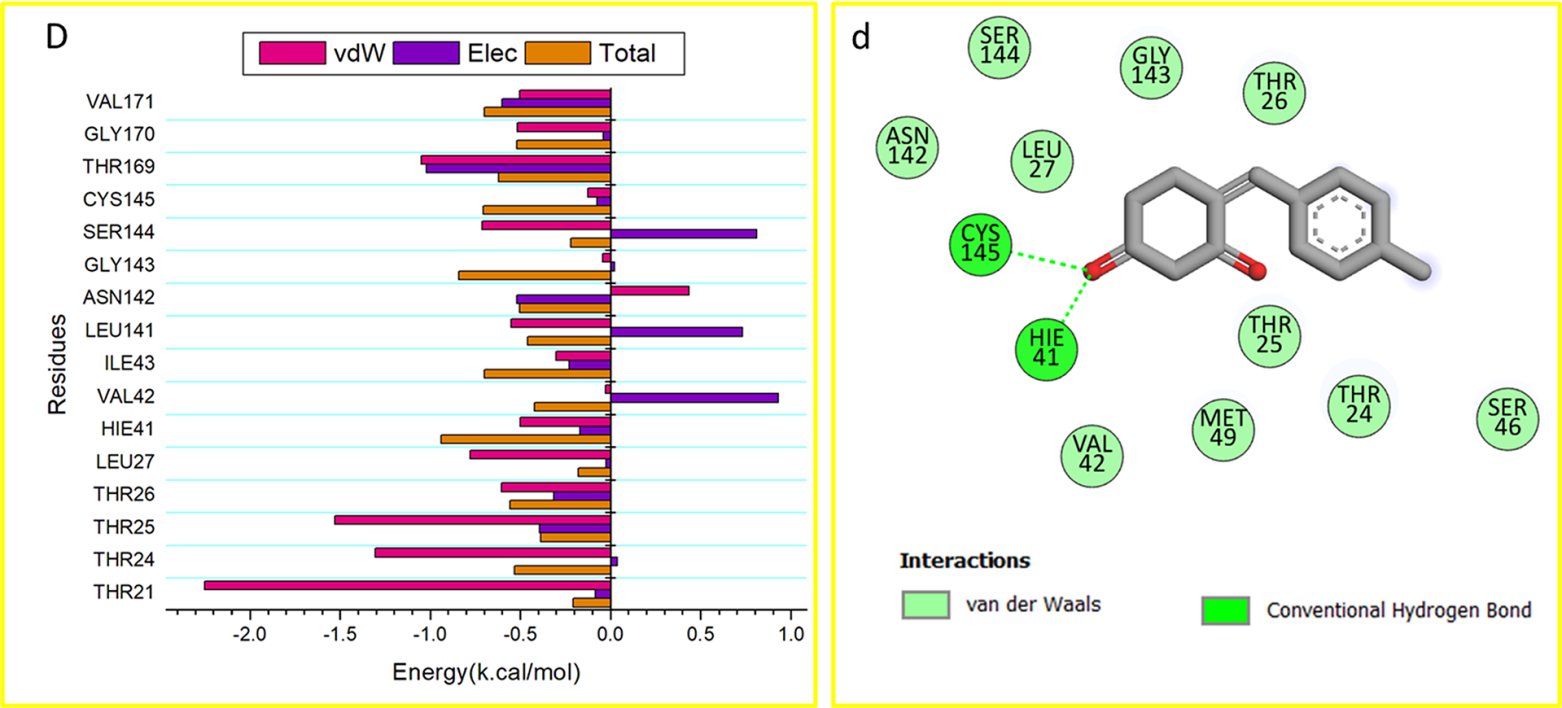
**(a)** [A] RMSD of Cα atoms of the protein backbone atoms. [B] RMSF of each residue of the protein backbone Cα atoms of protein residues ; (C) ROG of each protein residue’s Cα atoms; (D) solvent accessible surface area (SASA) of the Cα atoms relative (black) to the starting minimised over 100 ns for the main protease (M^pro^) of SARS-CoV-2 protein with ligand Hexadecanoic acid (red), Kojic acid(green) , Octanoic acid(blue), 4(4Methylbenzylidene) cyclohexane-1,3-dione (Cyn). **(b)** PCA projection of Cα atom motion constructed by plotting the first two principal components (PC1 and PC2) in conformational space, apo (black ), Hexadecanoic acid (red), Kojic acid(blue) , Octanoic acid(pink), and 4(4-Methylbenzylidene) cyclohexane-1,3-dione (green) , respectively. **(c)** Dynamic cross-correlation matrix analyses for main protease enzyme Apo (A), Kojic acid(B) Hexadecanoic acid (C), Octanoic acid(D), and 4(4Methylbenzylidene) cyclohexane-1,3-dione (E). Numbers closer to 1 indicate high correlation, while those closer to -1 indicate anticorrelation between pairs of residues. X denoted the binding site region of the main protease of SARS-CoV-2 (M^pro^) protein. **(d)** Conformational free energy landscape for Apo [A], Hexadecanoic acid [B], Kojic acid [C], Octanoic acid [D], 4(4-Methylbenzylidene)cyclohexane-1,3-dione [E] systems. **(e)** Per-residue decomposition plots showing the energy contributions to the binding and stabilization at the catalytic active site of the main protease of SARS-CoV-2 (M^pro^) protein [A] Hexadecanoic acid, [B] Kojic acid,[C] Octanoic acid, [D] 4(4Methylbenzylidene) cyclohexane-1,3-dione.

This study assessed Root-Mean-Square Deviation (RMSD) to measure the system’s stability during the 100 ns simulations. The recorded average RMSD values for all frames of systems apo-protein, Hexadecanoic acid-complex system, Kojic acid-complex system, Octanoic acid-complex system, and 4-(4-Methylbenzylidene) cyclohexane-1,3-dione system were 2.26 ± 0.20 Å, 1.89 ± 0.35 Å, 1.35 ± 0.18 Å, 2.185 ± 0.37 Å, and 1.90 ± 0.38 Å, respectively. (Fig. 5a). These results revealed that the Kojic acid-bound to protein complex system acquired a relatively more stable conformation than the other studied systems.

In MD simulation, checking how flexible the protein structure is when a ligand binds is important for looking at residue behavior and how it connects with the ligand^37^.

The Root-Mean-Square Fluctuation (RMSF) algorithm was used to look at changes in protein residues and see what happened when inhibitors bound to their targets over 100 ns of simulations. The computed average RMSF values were 3.89 ± 1.32 Å, 1.16 ± 0.49 Å, 1.15 ± 0.64 Å,1.27 ± 0.62 Å, and 1.42 ± 0.88 Å for apo-protein, Hexadecanoic acid-complex system, Kojic acid-complex system, Octanoic acid-complex system, and 4(4-Methylbenzylidene)cyclohexane-1,3-dione system respectively. Overall residue fluctuations of individual systems are represented in (Fig. 5a). These values revealed that the Kojic acid-bound to protein complex system has a lower residue fluctuation than the other system inhibition.

ROG was found to be a valuable tool for evaluating the stability and overall compactness of the system during MD simulation^38,39^. The average Rg values were 22.37 ± 0.1 Å, 22.25 ± 0.10 Å, 22.20 ± 0.11 Å ,22.324 ± 0.117 Å, and 22.26 ± 0.10 Å for apo-protein, Hexadecanoic acid-complex system, Kojic acid-complex system, Octanoic acid-complex system, and 4(4-Methylbenzylidene)cyclohexane-1,3-dione system respectively (Fig. 5a). The Kojic acid -bound complex has a very rigid structure against the major the main protease of SARS-CoV-2 (M^pro^) protein , according to the observed behavior.

The compactness of the protein’s hydrophobic core was assessed by calculating its solvent-accessible surface area (SASA). The stability of biomolecules was assessed by measuring the protein surface area visible to the solvent^40^. According to (Fig. 5a), the average SASA values were 14,517.16 Å, 14,212.52 Å, 14,018.26 Å, 14,501.67 Å, and 14,363.19 Å for apo-protein, Hexadecanoic acid-complex system, Kojic acid-complex system, Octanoic acid-complex system, and 4(4-Methylbenzylidene)cyclohexane-1,3-dione system, respectively (Fig. 5a). The Kojic acid- complex system is still intact inside the primary protease (M^pro^) binding site, according to the SASA discovery and the results of the RMSD, RMSF, and ROG simulations.

*Principal Component Analysis (PCA).*The PCA plot, shown in (Fig. 5b), shows clear and complex waves of conformation along the two main components in a significant subspace. There was a clear separation of motion between the apo-protein, Hexadecanoic acid-complex system, Kojic acid-complex system, Octanoic acid-complex system, and 4(4-Methylbenzylidene)cyclohexane-1,3-dione systems. As a result, the computed eigenvector from 100-ns MD trajectories for the three systems is pretty variable, showing how the proteins moved differently between the systems.

Since the apo system has more atomic fluctuations than the apo-protein, Hexadecanoic acid-complex system, Kojic acid-complex system, Octanoic acid-complex system, and 4(4-Methylbenzylidene)cyclohexane-1,3-dione system, it is likely that the ligand binding to the active sites of the protein, Hexadecanoic acid, Kojic acid-complex system, Octanoic acid-complex system, and 4(4-Methylbenzylidene)cyclohexane-1,3-dione system causes conformational dynamics, which is then reflected by the PCs as a wave of motion. The Kojic acid-complex system, on the other hand, is more densely packed than the other system. This means that when Kojic acid binds to the protein, it makes the protein less flexible, which makes it easier for ligands to bind to the active site.

DCCM analysis was performed on the position of Cα during the simulations to investigate the dynamics and existence of correlated motions, as shown in (Fig. 5c), in order to evaluate conformational changes of the main protease of SARS-CoV-2 (M^pro^) protein, following ligand interaction. Yellow–red (colour) regions indicate strongly positive-correlated motions of certain residues, while blue-black (colour) regions indicate highly negative-correlated motions of specific residues.

The studied systems showed that the residue’s overall correlated motions in comparison to anti-correlated motions. A study of DCCM says that when Kojic acid binds to the main protease protein, it gives the protein structural dynamics. This gives the protein conformational changes that are shown in the correlated motions.

Moreover, the binding site region (1–150 residues) showed a highly correlated motion in the kojic acid system in comparison to the other studied systems. Therefore, the potency of kojic acid as a main protease inhibitor may be a life dream compared to other systems that have demonstrated the characteristics of a highly potent agent on the main protease of the SARS-CoV-2 (M^pro^) protein .

### Free energy landscape

To investigate the effect of Hexadecanoic acid , Kojic acid , Octanoic acid ,and 4(4-Methylbenzylidene)cyclohexane-1,3-dione on the miss-folding of the main protease of SARS-CoV-2 (M^pro^) protein , we have plotted two- dimensional free energy contour maps as a function of RMSD and RG for Hexadecanoic acid , Kojic acid , Octanoic acid ,and 4(4-Methylbenzylidene)cyclohexane-1,3-dione systems with representative structures at each local free energy basin (Fig. 5d). In the Hexadecanoic acid , Kojic acid , Octanoic acid ,and 4(4-Methylbenzylidene)cyclohexane-1,3-dione systems, there was a sizeable conformational space compared to the Apo system (Fig. 5d). Overall, this data has shown that Hexadecanoic acid , Kojic acid , Octanoic acid ,and 4(4-Methylbenzylidene)cyclohexane-1,3-dione affected the free energy landscape of the main protease of SARS-CoV-2 (M^pro^) conformation.

### Binding interaction mechanism based on binding free energy calculation

The molecular mechanics energy technique (MM/GBSA) is often used to find the free binding energies of small molecules to biological macromolecules. It combines the generalized Born and surface area continuum solvation and may be more reliable than docking scores^41^. The MM-GBSA program in AMBER18 was used to calculate the binding free energies by extracting snapshots from the trajectories of the systems. As shown in Table 3, all the reported calculated energy components (except ΔG_solv_) gave high negative values, indicating favorable interactions. The results indicated that the binding affinity of Hexadecanoic acid -bonded to protein was -26.87 kcal/mol, Kojic acid bound to protein was -34.01 kcal/mol, the binding affinity of Octanoic acid bonded to protein was -14.97 kcal/mol, and the binding affinity of 4(4-Methylbenzylidene) cyclohexane-1,3-dione bonded to protein was -12.69 kcal/mol.

**Table 3:** Shows the calculated energy binding for the identified compounds against the main protease of SARS-CoV-2 (M^pro^) protein**.**

Energy Components (kcal/mol)						
Complex	ΔE_vdW_	ΔE_elec_	ΔG_gas_	ΔG_solv_	TΔS	ΔG_bind_
Hexadecanoic acid	-33.09 ± 0.25	-3.72 ± 0.38	-36.82 ± 0.47	9.94 ± 0.29	-16.23 ± 0.18	-26.87 ± 0.26
Kojic acid	-35.88 ± 0.22	-18.12 ± 0.79	-15.96 ± 0.37	18.04 ± 0.49	-21.11 ± 0.41	-34.01 ± 0.21
Octanoic acid	-6.82 ± 0.27	-2.72 ± 0.60	-9.55 ± 0.51	4.58 ± 0.89	-14.23 ± 0.30	-14.97 ± 0.53
4(4-Methylbenzylidene)- cyclohexane-1,3-dione	-7.48 ± 0.33	-2.95 ± 0.19	-10.44 ± 0.49	5.74 ± 0.26	-10.21 ± 0.26	-12.69 ± 0.24

∆EvdW = van der Waals energy; ∆Eele = electrostatic energy; ∆Gsolv = solvation free energy; ∆Gbind = calculated total binding free energy; ΔTS = ∆Gbind = calculated total binding free energy, TΔS (entropy contribution term), T = temperature (298.15 K).

The binding free energy is challenging to calculate in silico because of the challenge in entropy estimation. The solution to all possible solvent conformations and solute entropy requires computing the range of values of the underlying degree of freedoms corresponding to different conformations with the same potential energy (Table 3)^42^. The estimates of the interaction entropy changes during the formation of Hexadecanoic acid—M^pro^, Kojic acid—M^pro^, Octanoic acid—M^pro^ and 4(4-Methylbenzylidene)-cyclohexane-1,3-dione—M^pro^ complexes were obtained from a normal mode analysis of the energy-minimized structures. The average entropy terms obtained from the analysis were translational (-12.05 ± 0.0) kcal/mol, rotational (-11.92 ± 0.02) kcal/mol and vibrational (6.24 ± 0.58) kcal/mol for Hexadecanoic acid—M^pro^, while translational (-12.32 ± 0.0) kcal/mol, rotational (-12.45 ± 0.004) kcal/mol, and vibrational (5.61 ± 0.40) kcal/mol were recorded for Kojic acid—M^pro^, translational (-10.23 ± 0.01) kcal/mol, rotational (-9.72 ± 0.12) kcal/mol and vibrational (5.24 ± 0.48) kcal/mol for Octanoic acid—M^pro^ ,and translational (-9.85 ± 0.02) kcal/mol, rotational (-8.45 ± 0.003) kcal/mol, and vibrational (4.81 ± 0.30) kcal/mol were recorded for (4-Methylbenzylidene)- cyclohexane-1,3-dione—M^pro^ Complex. The conformational entropy TΔS were -16.23 ± 0.18 kcal/mol , -21.11 ± 0.41 kcal/mol , -14.23 ± 0.30 kcal/mol, -10.21 ± 0.26 kcal/mol for Hexadecanoic acid—M^pro^, Kojic acid—M^pro^ , Octanoic acid—M^pro^ and 4(4-Methylbenzylidene)- cyclohexane-1,3-dione—M^pro^ complexes, respectively.

The interactions between the ligands that were extracted and the main protease (M^pro^) of the SARS-CoV-2 protein residues are governed by the more positive Van der Waals energy components. This was shown by a close study of each energy contribution, which led to the reported binding free energies (Table 3).

### Identification of the critical residues responsible for ligand binding

We broke down the total energy needed for the identified compounds to bind to this enzyme into the energy involved at each site residue level to learn more about the important residues that stop the main protease (M^pro^) of the SARS-CoV-2 protein from working.

From (Fig. 5e), the primary positive impact of Hexadecanoic acid compound on the main protease of SARS-CoV-2 (M^pro^) protein is predominantly observed from residues Thr 25 (-1.199 kcal/mol), Thr 26(- 0.263 kcal/mol), Leu 27 (-0.666 kcal/mol), HIE 41 (-0.763 kcal/mol), Val 42 (-0.204 kcal/mol), Cys 44 (-0.416 kcal/mol), Thr 45 (-0.534 kcal/mol), Ser 46 (-0.993kcal/mol), Met 49 (-1.089 kcal/mol), Phe 140 (-0.355kcal/mol), Leu 141 (-0.57 kcal/mol), Asn 142 (-0.208kcal/mol), Ser 144( -0.496 kcal/mol), Cys 145(-0.923 kcal/mol), HIE 163(-0.381 kcal/mol), Met 165 (-0.683kcal/mol) and HIE 172 (-0.266kcal/mol).

In contrast, the significant favorable impact of the Kojic acid compound the main protease of SARS-CoV-2 (M^pro^) protein is predominantly observed from residues Thr 26 (-1.243 kcal/mol), Leu 27 (-0.855 kcal/mol), Asn 28 (-0.138 kcal/mol), Met 49 (-0.647 kcal/mol), Asn 142 (-1.043 kcal/mol), Gly 143(-1.174 kcal/mol), Ser 144( -0.81 kcal/mol), Cys 145(-1.429 kcal/mol), and HIE 163(-0.103 kcal/mol).

The significant favorable impact of Octanoic acid on the main protease of SARS-CoV-2 (M^pro^) protein is predominantly observed from residues Thr 25 (-0.428 kcal/mol), Leu 27 (-0.859 kcal/mol), Thr 45 (-0.378 kcal/mol), Ser 46(-0.736 kcal/mol), Met 49 (-0.891 kcal/mol), Asn 142 (-0.982 kcal/mol),cys 145(-0.77 kcal/mol), Gly 146 ( -0.82 kcal/mol), and Ser 147(-0.652 kcal/mol).

Finally, the significant favorable impact of 4(4-Methylbenzylidene)cyclohexane-1,3-dione compound on the main protease of SARS-CoV-2 (M^pro^) protein is predominantly observed from residues THR24 (-1.03 kcal/mol), Thr 25 (-1.528 kcal/mol), Thr 26 (-0.6 kcal/mol), Leu 27 (-0.779 kcal/mol), His 41 (-0.501 kcal/mol), Ile 43 (-0.302 kcal/mol), Leu 141 (-0.552 kcal/mol), Asn 142 (-0.437 kcal/mol), Ser 144 (-0.713 kcal/mol), Cys 145 (-0.126 kcal/mol), Thr 169 ( -1.05 kcal/mol), Gly 170 (-0.515 kcal/mol), and Val 171 (-0.504 kcal/mol).

## Conclusion

The COVID-19 pandemic has resulted in the loss of over one million mortalities and impacted millions of individuals. Mainly, the severe acute respiratory syndrome Corona Virus-2 (SARS CoV-2) has the potential to induce life-threatening diseases.

The fungal extract and its primary constituent, kojic acid (65%), demonstrated effective eradication of the highly pathogenic SARS-CoV-2. Compared to Remdesivir, which was effective at treating SARS-CoV-2 with medicine, A. tamarii extract was not very successful at killing human Adeno-7-Virus and Herpes-2 virus.

Because co-infecting microorganisms may contribute to respiratory and immune system impairment, the extract is potentially effective against gram-negative bacteria (E. coli and P. aeruginosa).

There were 25 compounds found through GC/MS analysis. The molecular docking study showed that four of them had a good chance of blocking the SARS-CoV-2 protein. These compounds exhibited a docking score higher than Ramsidiver (− 9.89 kcal/mol) (Fig. 4). We studied the dynamic behavior of the four best-docked protein–ligand complexes on a time scale of 100 ns, based on their respective RMSD and RMSF.

**Fig. 6:**
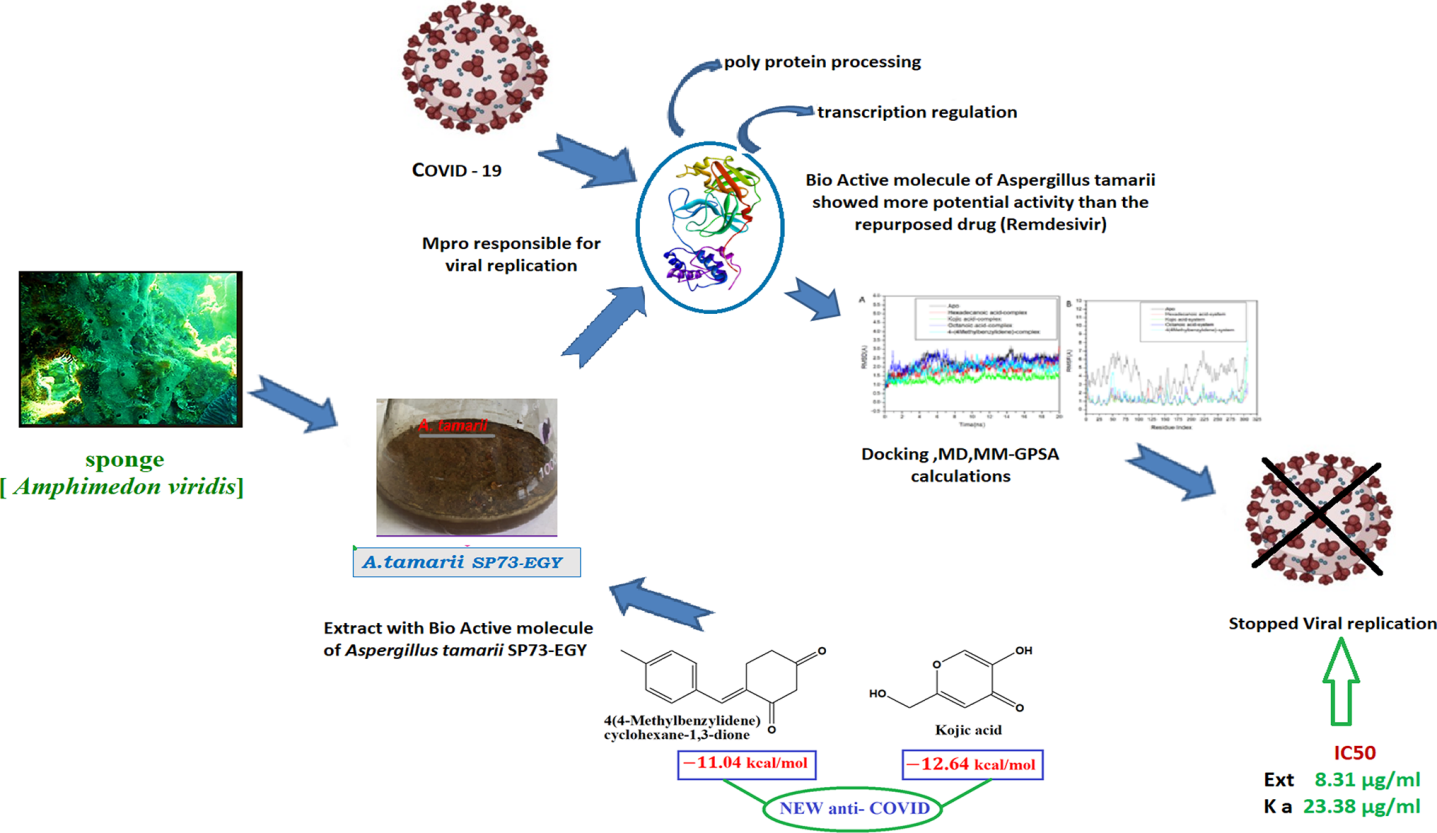
Conclusive graphical abstract: The fungus was isolated from the sponge *Amphimedon viridis*. The fungal extract showed significant antiviral activity against COVID-19 (SARS-CoV-2 virus). Bio-active molecules of *Aspergillus tamarii* showed more potential activity than the repurposed drug (Remdesivir) through Docking, MD and MM-GPSA calculations. New anti-COVID-19 compounds were identified and isolated.

Finally, for the first time, the study investigated the highly significant anti-COVID-19 impact of *Aspergillus tamarii* isolate SP73-EGY extract "derived from the sponge *Amphimedon viridis*, Red Sea, Egypt." In-vitro and molecular docking experiments supported the study. Moreover, we discovered new anti-SARS-CoV-2 in the major isolated compound, "kojic acid," and the identified compound, "4(4-Methylbenzylidene)cyclohexane-1,3-dione" (Fig. 6).

The results showed that the fungal extract from *Aspergillus tamarii* isolate SP73-EGY was effective at fighting the virus SARS-CoV-2 in both in-vitro and in silico tests. Future studies on toxicity, bioaccessibility, bioavailability, and animal models could utilize this fungus extract as a starting point for commercialization.

## Methods

General. The UV spectrum was recorded in MeOH on a UVIDEC-610C double-beam spectrophotometer (JASCO). IR spectrum was recorded on an FTIR spectrometer Perkin-Elmer 1605 using KBr discs as a beam splitter. Electron impact mass spectrometry (EI-MS) analysis involves turning a compound into a gas in a chamber that is otherwise empty and then hitting it with electrons that are 25–80 e (2.4–7.6 MJ/mol) strong. A Bruker DRX 400 spectrometer (BrukerBiospin, Rheinstetten, Germany) was used to record the NMR spectra in DMSO-d6 as the solvent. ^1^H and ^13^C-NMR, HSQC, COSY, andHMBC spectra were measured using an inverse-detection probe (5 mm). Operating frequencies were 400 MHz for acquiring ^1^H-NMR and 125 MHz for ^13^C-NMR spectra. Chemical shifts were given in δ (ppm) relative to TMS.

### Collection of sponge materials

The sponge *Amphimedon viridis* was gathered from Hurghada, Egypt. The geographical coordinates of Shaa’b Al Areq are located at a latitude of N 27° 25′ 08.9 and a longitude of E 33° 51′ 0.5, situated along the coastline of the Red Sea. The specimen was extracted at a depth ranging from 5 to 8 m and subsequently frozen for further examination. The anatomical taxonomy of sponges was revised by Mohamed A. Ghani, an ecological researcher affiliated with the Red Sea Marine Parks in Hurghada, Egypt.

### Preparation of sponge material and isolation of *fungi*

The process of isolating fungi from sponges entailed aseptically homogenizing the samples and subsequently diluting the resulting homogenate. Each dilution was then used to inoculate the appropriate fungal isolation media. The inoculated media were kept at 30 °C for 7 to 14 days before the colonies of pure-appearing fungi were removed^43^.

### Screening medium

The fungus *Aspergillus tamarii* isolate SP73-EGY was cultivated in a medium consisting of potato dextrose broth supplemented with 20 g/l of dextrose and 200 chipped potatoes infusion. A total of eight conical flasks, each with a volume of 1 L, were filled with 200 ml of broth medium. These flasks were then inoculated with spore suspensions obtained from the fungus slant. The cultivated flasks were incubated at a temperature of 30 °C for a period of 12 days with no agitation^43^.

### Preparation of fungal extract

Using a cooling centrifuge, the fungal culture was first centrifuged at 8,000 rpm at 4 °C to remove the mycelia (Centurion Scientific Ltd model K2015R, UK). The culture supernatant was extracted with ethyl acetate (EtOAc) and dried under vacuum^18^.

### Molecular identification of selected fungal strain SP73-EGY

Fungal culture was identified according to a molecular biological protocol by DNA isolation, amplification (PCR) and sequencing of the ITS region. The primers ITS2 (GCTGCGTTCTTCATCGATGC) and ITS3 (GCATCGATGAAGAACGCAGC) were used at PCR while ITS1 (TCCGTAGGTGAACCTGCGG) and ITS4 (TCCTCCGCTTATTGATATGC) were used at sequencing. The purification of the PCR products was carried to remove unincorporated PCR primers and dNTPs from PCR products by using Montage PCR Clean up kit (Millipore). Sequencing was performed by using Big Dye terminator cycle sequencing kit (Applied BioSystems, USA). Sequencing products were resolved on an Applied Biosystems model 3730XL automated DNA sequencing system (Applied BioSystems, USA). *Candida* sp. was used as control^43^.

### GC/MS) analysis

Dry extract (1.5 mg) and 20 μl pyridine were mixed well; then, 30 μl of N,O-bis-(trimethylsilyl) trifluoroacetamide was added and heated at 80 °C for 30 min. Finally, GC/MS analysis was performed^44^.

A mass spectrometer (Finnigan MAT SSQ 7000) was attached to a Varian 3400 gas chromatograph. The DB-5 column had an internal diameter of 30 m × 0.32 mm; the carrier gas was helium (He pressure, 20 Mpa/cm2); 310 °C was the injector temperature; 85–310 °C at 3 °C/minute (10 min initial hold) was GC temperature program; and EI mode was at 70 eV. The scan repetition rate was 0.5 s over a mass range of 39–650 amu.

Identification was based on computer searches of user-generated reference libraries (mainlib and Wiley9) that included mass spectra. Peaks were validated for homogeneity using single-ion chromatographic reconstruction^18^.

### Isolation of the major compound identified by GC/MS analysis

The ethyl acetate extract underwent GC/MS analysis, which identified the existence of a significant component (65%). A crystalline needle substance was produced after the extract was repeatedly crystallized by methanol and water.

### Structure Elucidation of kojic acid

We used mass spectrometry (EI-MS), UV, IR, EDX, and 1D- and 2D-NMR spectroscopy to figure out the structure of the compound that was isolated (Table 2). The APT experiment distinguished between methyl, methylene, and methine carbon atoms. The COSY method provides data on homonuclear ^1^H-^1^H scalar couplings. Modifying the HSQC gradient pulse parameters allowed for the identification of 1H-13C one-bond connectivity. The HMBC experiment detected connections between two and three bonds. Chemical shifts have been observed in δ(ppm).

### Antiviral assay

#### Cytotoxicity assay to determinate cytotoxic concentration (CC_50_)

In brief, cells were seeded into a 96-well culture plate at a density of 2 × 10^4^ cells/well one day before infection. The culture medium was removed the next day, and the cells were washed with phosphate-buffered saline. The cells were treated with 100 μL from extracts with a concentration of 1000 µg/ml as a start concentration and serial dilution two-fold for ten dilutions and the last two dilutions have been cell control without treatment each dilution was made in triplicate form. Cells were grown in DMEM medium supplemented with 10% fetal bovine serum and 0.1% antibiotic/antimycotic solution. Gibco BRL provided the antibiotic and antimycotic solution, trypsin–EDTA, fetal bovine serum, and DMEM medium (Grand Island, NY, USA. The cell monolayers were fixed with 10% formaldehyde for 72 h and stained with a 0.03% crystal violet solution in 2% ethanol. After washing and drying, the crystal violet dye was then dissolved using 100 μL absolute methanol per well, and the optical density of the color was measured at 570 nm using an Anthos Zenyth 200rt plate reader (Anthos Labtec Instruments, Heerhugowaard, Netherlands). The IC_50_ of the compound is required to reduce the virus-induced cytopathic effect (CPE) by 50% relative to the virus control. The optical density of individual wells was quantified spectrophotometrically at 570/630 nm^45^.

The cytotoxic concentration (CC50) was calculated by using GraphPad PRISM software (Graph-Pad Software, San Diego, USA).

### Antiviral assay to determination of inhibitory concentration (IC_50_)

A test called cytopathic effect (CPE) inhibition assay was used to find possible antivirals that could fight the highly pathogenic coronavirus SARS-CoV-2, the low pathogenic coronavirus (229E), herpes-2, and Adeno-7 viruses. The dose–response assay was made to find the range of how well the chosen antiviral worked (IC50) and how harmful it was to cells (CC50). This assay is critical for determining antiviral efficacy in cell culture systems. The biosafety levels used to handle the pathogenic cultures were level three.

To test SARS-CoV-2, Low Pathogenic Corona Virus (229E), Herpes-2 virus, and Adeno-7, Vero E6 cells, Vero cells, and Hep-2 cells were used, in that order. Cells were grown in DMEM medium supplemented with 10% fetal bovine serum and 0.1% antibiotic/antimycotic solution. Gibco BRL provided the antibiotic and antimycotic solution, trypsin–EDTA, fetal bovine serum, and DMEM medium (Grand Island, NY, USA). The Crystal Violet method was used to evaluate antiviral activity using the recently reported cytopathic (CPE) inhibition cells were infected with 100 µl from the diluted virus according to TCID_50_ of each virus. The dilution will be prepared. In this assay, the dilution of viruses was 10^–4.^ This dilution was suitable to produce the desired CPEs after three days post-infection and incubate for 1 h with serial dilutions. Twofold of the extract started with a safe dose before addition to the cell line—the virus controls (virus-infected, nondrug-treated cells) and cell controls (non-infected, nondrug-treated cells). The cell monolayers were fixed with 10% formaldehyde for 72 h and stained with a 0.03% crystal violet solution in 2% ethanol. After the samples were washed and dried, 100 μL of absolute methanol was used to dissolve the crystal violet dye. The optical density of the color was then measured at 570 nm using an Anthos Zenyth 200rt plate reader (Anthos Labtec Instruments, Heerhugowaard, Netherlands). The IC_50_ of the compound is required to reduce the virus-induced cytopathic effect (CPE) by 50% relative to the virus control. The optical density of individual wells was quantified spectrophotometrically at 570/630 nm^45^**.**

The selectivity index was calculated as follows:

$${\text{Selectivity}}\;{\text{ Index}}\left( {{\text{SI}}} \right) \, = {\text{estimated}}\;{\text{CC}}_{50} /{\text{estimated}}\;{\text{IC}}_{50}$$

The results of the 50% cytotoxic concentrations (CC_50_) and the 50% inhibitory concentration (IC_50_) were determined using GraphPad PRISM software (Graph-Pad Software, San Diego, USA).

### Antimicrobial assay

The cup-plate method was used to evaluate the fungal extract’s antimicrobial effects on *Staphylococcus aureus* ATCC 6538 (G + ve bacteria), *Pseudomonas aeruginosa* ATCC 9027, *Escherichia coli* ATCC 8739 (G-ve bacteria), and *Candida albicans* ATCC 10,231 (yeast)^46^. The produced nutrient agar (NA) plate was inoculated with the test organism with a thickness of 4–5 mm and then allowed to harden. The (NA) dish should be split into four equal pieces. Then, four cavities were created in each part using a sterile borer. The extract is then put into four cavities at various concentrations (ranging from 25 to 200 mg/ml). Next, let the plates gently incubate for 24.0 + /- 2.0 h at 37 °C. *Candida albicans* were incubated for 48.0 ± 2.0 h.

The inhibition zone was determined using the equation X = a—b / 2.

Where "a" is the diameter of the inhibition zone and "b" is the diameter of the well (10 mm).

*Achievement of microorganisms and experiments were by Nawah-scientific.

### Molecular docking analysis

Molecular docking protocols are widely used for predicting the binding affinities for a number of ligands. In the current work, we aimed to examine the possibility of an existing relationship between the experimental bioactivities of the inhibitors under study and the docking scores. In order to get accurate results, all the docking experiments were performed with the default parameters. The duration required to dock one ligand ranged approximately from 1–2 min. The docking procedure was conducted according to the literature^46^.

### Molecular dynamic and system stability

*System preparation.* The crystal structure of the main protease (M^pro^) of SARS-CoV-2, solved at a resolution of 2.1 Å, was retrieved from the protein data bank with codes 6LU7^48^. The given structure was successfully resolved with a resolution of 2.1. Then, using UCSF Chimera, these structures were produced for molecular dynamics (MD) experiments^49^. pH was fixed and tuned to 7.5 using PROPKA^50^. ChemBioDraw Ultra 12.1 was used to illustrate the structures of hexadecanoic acid, octanoic acid, kojic acid, and 4(4-Methylbenzylidene)- cyclohexane-1,3-dione^51^. All two prepared systems were run through 100 ns MD simulations per the simulation section’s instructions.

### Molecular dynamic (MD) simulations

Molecular dynamic (MD) simulations are used to study biological systems and can be used to look into how atoms and molecules move physically 45. This simulation’s insight offers a complex perspective on the dynamical evolution of biological systems, including conformational changes and molecular interaction^50^. The PMEMD engine’s GPU version, part of the AMBER 18 package^51^, was used to run MD simulations on all systems. The Molecular dynamic procedure was conducted according to the literature^47^.

### Principal component analysis

PCA is a multivariate statistical technique, applied to systematically reduce the number of dimensions needed to describe the protein dynamics^52^, through the decomposition process that screen observed motions from largest to smallest spatial scale^50^. Using PCA, you can figure out how the atomic positions and shapes of proteins change by taking different modes of the protein complex’s shape during simulations of its dynamics. The direction of motion (eigenvectors) and the extent of motion (eigenvalues) of the biological system can also be determined using PCA^52^. Herein, 100 ns of MD trajectories was stripped of the solvent molecules and the ions using the CPPTRAJ module in Amber14^52^^.^This was done prior to MD trajectory processing for PCA. PCA was performed on Cα atoms for 1000 snapshots at 100-ps time interval each using in-house scripts. The first two principal components (PC1 and PC2) were computed and 2 × 2 covariance matrices were generated using Cartesian coordinates of Cα atoms. PC1 and PC2 correspond to first two eigenvectors of a covariant matrix. Origin software was used to construct the PC plot.

### 2D free energy surfaces (2DFESs)

The computation of 2DFESs has been performed as follows: A pair of metrics were computed from each snapshot : the Root Mean Square Deviation (RMSD) of the conformation in that snapshot compared to the conformation of the extracted compounds , and the Radius of Gyration (Rg) of the extracted compound while coupled to the main protease (Mpro) of SARS-CoV-2 (at the Cα atom level). These were classified into bins to provide two-dimensional histograms. The determination of the 2DFES involved the computation of the negative logarithms of the 2D-histogram values (-ln (populations)).

### Dynamic cross-correlation matrices (DCCM)

The cross-correlation is a 3D matrix representation that visually depicts information about time correlations between protein residues^53^. Visual pattern recognition can be used to examine data that is based on residue and is time correlated^53^. A DCCM was produced using the following equation to find the cross-correlated displacements of backbone C-α atoms in the trajectories, in order to better understand the dynamics of apo-protein, Hexadecanoic acid-complex system, Kojic acid-complex system, Octanoic acid-complex system, and 4(4-Methylbenzylidene)cyclohexane-1,3-dione system:

1 $$C_{ij} = \frac{{ < \Delta {\text{ri}} \cdot \Delta {\text{rj > }}}}{{( < \Delta {\text{r}}_{{\text{i}}}^{2} > < \Delta {\text{r}}_{{\text{j}}}^{2} > )^{\frac{1}{2}} }}$$

where Δ ri is the displacement of the ith Cα aom relative to its averaged position. The Δri is the displacement of the ith Cα atom relative to its averaged position. Significantly correlated movements are symbolized by Cij = 1, while Cij = -1 symbolized highly anticorrelated movements in the trajectory. The divergence of motion from 1 and -1 indicates that i and j movements are anticorrelated.

The DCCM matrix was carried out using the CPPTRAJ package in Amber 18, and the matrices were plotted and evaluated using Origin software (www.originlab.com).

*Thermodynamic calculation.* The utilization of the Poisson-Boltzmann or generalized Born and surface area continuum solvation (MM/PBSA and MM/GBSA) technique has demonstrated its efficacy in the estimation of ligand-binding affinities^54,55^. The MMgGPSA calculation was conducted in accordance with the methodology described in the literature^47^.The interaction entropy (-TΔS) was estimated using 40 frames due to the computational cost of calculating the change in conformational energy for large frames using the normal mode method^56^. We found out how much each residue adds to the total binding free energy at the predicted active site by breaking down the atomic level energy per residue using the MM/GBSA method in AMBE 18^57,58^.

## Electronic Supplementary Material

Below is the link to the electronic supplementary material.

Supplementary Material 1

## Data Availability

The datasets used and/or analysed during the current study available from the corresponding author on reasonable request.
