# Supplementary material for: Combating COVID-19 and its co-infection by Aspergillus tamarii SP73-EGY using in vitro and in silico Studies
Source: Sci Rep. 2025 Jan 3;15:685. doi: 10.1038/s41598-024-77854-0 (PMC11698736; doi:10.1038/s41598-024-77854-0)
Supplement: Supplementary file 1 — Supplementary Material 1 [file 41598_2024_77854_MOESM1_ESM.docx]

| **ligands** | ***Hydrogen bonds between atoms of ligands and amino acids of receptor*** | | | | | **S- score**  (binding energy)  (kcal/mol) | **Ligand efficiency** |
| --- | --- | --- | --- | --- | --- | --- | --- |
|  | ligands  Atoms | Receptor | | Type | Distance (Å) |  |  |
|  |  | Atoms | Residues |  |  |  |  |
| propylene glycol | O 4691 | OG 342 | THR 24 | H-acc | 2.20 | -6.75 | -1.35 |
|  | O 4691 | OG 342 | THR 24 | H-don | 2.20 |  |  |
| D-Lactic acid | O 4691 | OG 342 | THR 24 | H-acc | 2.61 | -8.96 | -1.49 |
|  | O 4691 | OG 342 | THR 24 | H-don | 2.61 |  |  |
| Nicotinamide | H 4696 | O 2189 | LEU 141 | H-don | 1.34 | -7.63 | -0.847 |
| **Octanoic acid** | **O 4707** | **OG 342** | **THR 24** | **H-don** | **3.00** | **-11.16** | **-1.116** |
|  | O 4707 | OG 356 | THR 25 | H-don | 2.34 |  |  |
|  | O 4707 | OG 342 | THR 24 | H-acc | 3.00 |  |  |
|  | O 4707 | OG 356 | THR 25 | H-acc | 2.34 |  |  |
| succinic acid | O 4696 | OG 690 | SER 46 | H-don | 2.99 | -10.10 | -1.26 |
|  | O 4696 | OG 690 | SER 46 | H-acc | 2.99 |  |  |
| **Hexadecanoic acid** | **O 4684** | **OG 342** | **THR 24** | **H-don** | **2.11** | **-11.91** | **-0.66** |
|  | O 4684 | OG 342 | THR 24 | H-acc | 2.11 |  |  |
| Oleic acid | O 4730 | OG 356 | THR 25 | H-don | 2.71 | -10.91 | -0.544 |
|  | O 4730 | OG 356 | THR 25 | H-acc | 2.71 |  |  |
| 4-Hydroxybutanoic acid | O 4696 | OG 342 | THR 24 | H-don | 2.75 | -9.54 | -1.362 |
|  | H 4697 | O 339 | THR 24 | H-don | 3.01 |  |  |
|  | O 4698 | OG 356 | THR 25 | H-don | 2.87 |  |  |
|  | O 4684 | OG 675 | THR 45 | H-don | 2.99 |  |  |
|  | O 4684 | OG 690 | SER 46 | H-don | 2.67 |  |  |
|  | O 4696 | OG 342 | THR 24 | H-acc | 2.75 |  |  |
|  | O 4698 | OG 356 | THR 25 | H-acc | 2.87 |  |  |
|  | O 4684 | OG 675 | THR 45 | H-acc | 2.99 |  |  |
|  | O 4684 | OG 690 | SER 46 | H-acc | 2.67 |  |  |
| Diethylene glycol | O 4684 | OG 675 | THR 45 | H-don | 2.78 | -8.84 | -1.26 |
|  | O 4684 | OG 690 | SER 46 | H-don | 2.41 |  |  |
|  | O 4684 | OG 675 | THR 45 | H-acc | 2.78 |  |  |
|  | O 4684 | OG 690 | SER 46 | H-acc | 2.41 |  |  |
|  | O 4684 | N 681 | SER 46 | H-acc | 2.65 |  |  |
| 2,3-Dihydroxybutane | H 4697 | O 339 | THR 24 | H-don | 1.38 | -7.49 | -1.24 |
|  | O 4698 | OG 675 | THR 45 | H-don | 2.66 |  |  |
|  | O 4698 | OG 690 | SER 46 | H-don | 3.06 |  |  |
|  | O 4698 | OG 675 | THR 45 | H-acc | 2.66 |  |  |
|  | O 4698 | OG 690 | SER 46 | H-acc | 3.06 |  |  |
| 4-Chlorobenzoic acid | O 4697 | N 2235 | CYS 145 | H-acc | 2.65 | -9.26 | -0.926 |
| 2-isopropyl-3-hydroxy-2-  butenoic acid | H 4692 | O 2189 | LEU 141 | H-don | 3.60 | -9.73 | -0.973 |
|  | O 4691 | N 2217 | LEU 141 | H-don | 3.60 |  |  |
|  | O 4691 | N 2217 | GLY 143 | H-acc | 2.91 |  |  |
|  | O 4704 | N 2217 | GLY 143 | H-acc | 2.28 |  |  |
| Cyclohexanone-3-carboxylic  **acid** | O 4699 | OG 690 | SER 46 | H-acc | 2.49 | -10.21 | -0.12 |
| **4(4-Methylbenzylidene)**  **cyclohexane-1,3-dione** | **O 4713** | **OG 342** | **THR 24** | **H-acc** | **2.45** | **-11.04** | **-0.69** |
| **Kojic acid** | **O 4695** | **N 2217** | **GLY 143** | **H-acc** | **2.60** | **-12.64** | **-1.264** |
| N,N,N',N'-Tetramethyl-p-phenyl  phosphonic diamide | O 4696 | NE 619 | HIS 41 | H-acc | 2.65 | -8.13 | -0.45 |
| 1,11-Bis(methoxycarbonyl-ethenyl)-10,2-dihydroxy-cycloeicosane | O 4765 | OG 342 | THR 24 | H-acc | 2.69 | -10.74 | -0.315 |
|  | O 4759 | OG 2233 | SER 144 | H-acc | 2.15 |  |  |
| 2,3-Dihydroxy-Propanoic acid | O 4689 | NE 2506 | HIS 163 | H-acc | 2.69 | -7.97 | -1.138 |
| Valeric acid-5-methoxy | O 4693 | NE 2506 | HIS 163 | H-acc | 2.67 | -7.76 | -0.776 |
| 3,4-dihydroxy butanoic acid | O 4684 | NE 2506 | HIS 163 | H-acc | 2.70 | -7.87 | -0.98 |
| Azelaic acid | O 4694 | OH 816 | TYR 54 | H-acc | 2.77 | -5.39 | -0.414 |
|  | O 4696 | NE 2506 | HIS 163 | H-acc | 2.46 |  |  |
| stearic acid | O 4695 | OG 2233 | SER 144 | H-acc | 2.78 | -5.05 | -0.2505 |
| Benzoic acid-3,4-dimethoxy | O 4690 | N 2217 | GLY 143 | H-acc | 2.65 | -6.29 | -0.4838 |
| 2,4-Di(cyclopenta-2,4-dien-1-ylidene)1,1,3,3-tetramethyl- cyclobutane | C 4685 | NE 619 | HIS 41 | H-acc | 2.73 | -6.93 | -0.385 |
| 2-Aminoquinolino-[8,7e][1,2,4] triazine-4-oxide | H 4705 | O 2189 | LEU 141 | H-don | 2.07 | -5.27 | -0.3293 |
| **Remdesivir** | H 4697 | OG 356 | THR 25 | H-don | 0.94 | **-9.98** | **0.23** |
|  | O 4695 | OG 675 | THR 45 | H-acc | 2.13 |  |  |

**Table S1. Summary of Molecular Operating Environment (MOE) docking results for all the fungal extract identified compounds by GC/MS analysis with SARS-CoV-2 M^pro^ receptor.**


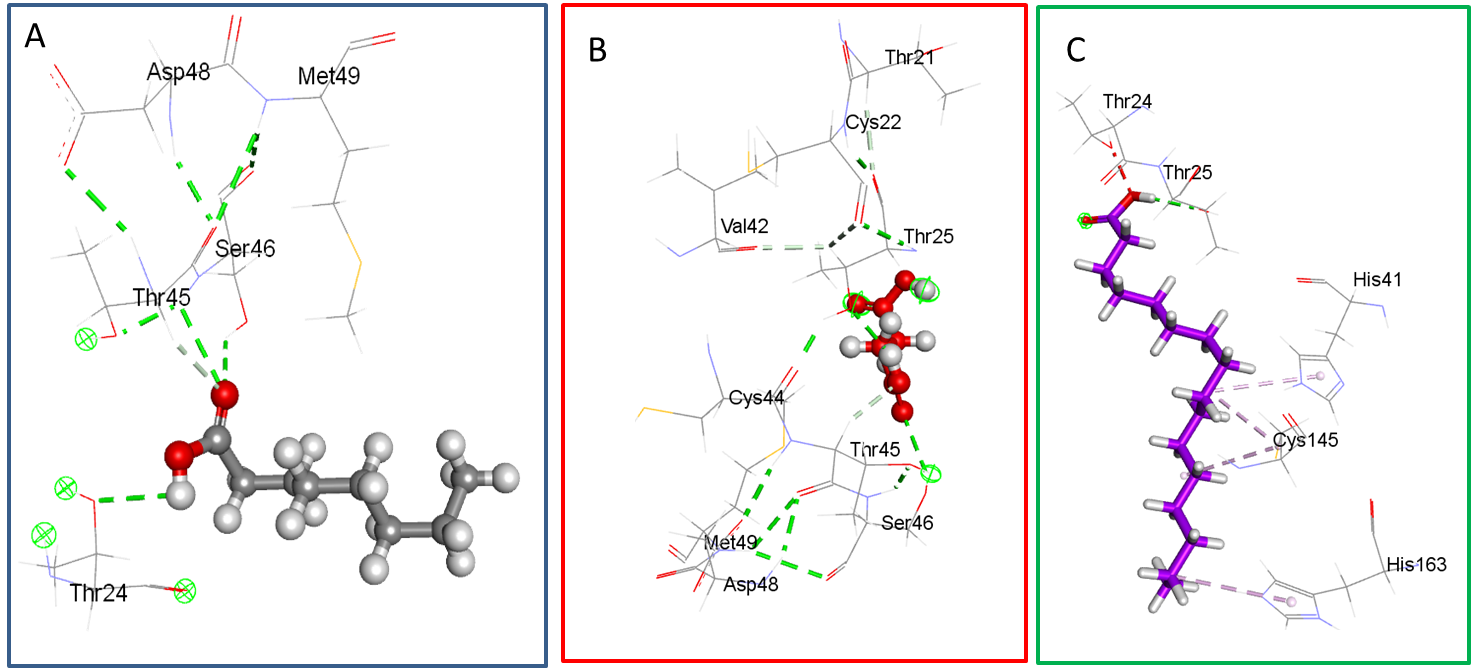


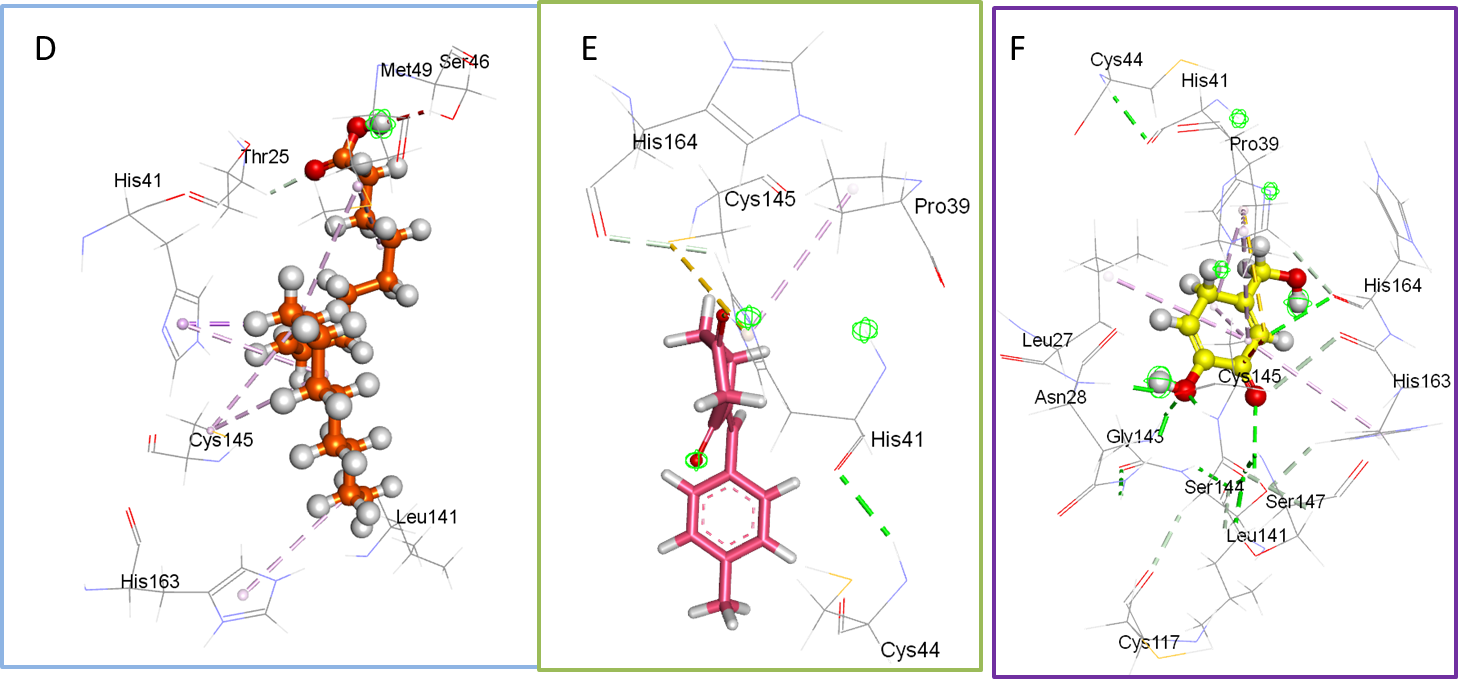


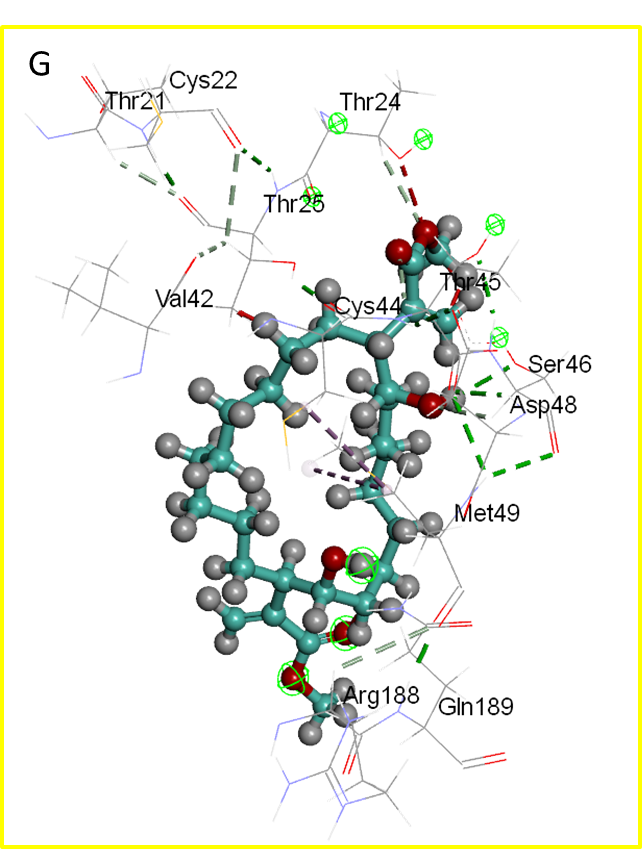


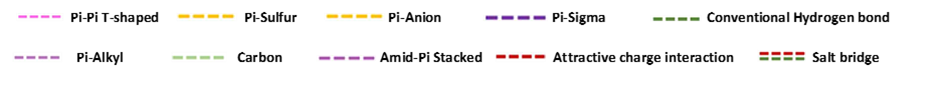


**Figure S1** : Inter-molecular interactions between A) **Octanoic** acid- SARS-CoV-2 M**^pro^** receptor B) succinic acid - SARS-CoV-2 M**^pro^** receptor C) **Hexadecanoic acid**- SARS-CoV-2 M**^pro^** receptor ,D) Oleic acid – SARS-CoV-2 M**^pro^** receptor ,E) **4(4-Methylbenzylidene)cyclohexane-1,3-dione** - SARS-CoV-2 M**^pro^** receptor , F) **Kojic acid** - SARS-CoV-2 M**^pro^** receptor, G) 1,11-Bis(methoxycarbonyl-ethenyl)-10,2-dihydroxy-cycloeicosane-SARS-CoV-2 M**^pro^** receptor.
